# Preliminary Study of the Antimicrobial Capacity of the Cutaneous Mucus and Smear Cytology of the Epidermis in a Population of European eels (*Anguilla anguilla,* Linnaeus 1758)

**DOI:** 10.3390/ani15121810

**Published:** 2025-06-19

**Authors:** Enrico Volpe, Sara Ciulli, Maria Morini, Laura Gentile, Antonio Casalini, Chiara Gentilezza, Luciana Mandrioli

**Affiliations:** Department of Veterinary Medical Sciences, Alma Mater Studiorum University of Bologna, 40064 Bologna, Italy; enrico.volpe2@unibo.it (E.V.); sara.ciulli@unibo.it (S.C.); maria.morini@unibo.it (M.M.); laura.gentile4@unibo.it (L.G.); antonio.casalini6@unibo.it (A.C.); chiara.gentilezza@studio.unibo.it (C.G.)

**Keywords:** antibacterial assay, *Aeromonas* sp., cytological analysis, epidermis, European eel, *Anguilla anguilla*, integument, mucus, skin

## Abstract

The skin and its products, such as the mucus, represent an important defense mechanism against infection by pathogens. Various environmental conditions can alter mucus composition and/or cells embedded in this matrix. The analysis of skin mucus is considered a useful method to evaluate the biological response of fish to stimuli. The mucus and cells can be considered suitable non-invasive biomarkers. With this preliminary study, an antibacterial capacity method was applied to cutaneous mucus samples of a European eel population. This standardized method highlighted the ability of the mucus to significantly counteract the proliferation of two bacteria, the opportunistic pathogen *Aeromonas hydrophila* and the primary pathogen *Aeromonas salmonicida*. The cytological method using skin scraping has proven to be a useful non-invasive tool, having allowed the highlighting of the two most represented cellular populations of the cutaneous covering, the epidermal cells and the goblet cells, in combination with mucous strands. Cytological findings did not disclose pathologic elements in the mucus. A broader use of these two non-invasive microbiological and cytological methods can provide useful information on fish health, indirectly contributing to the conservation of the species.

## Simple Summary

The European eel (*Anguilla anguilla*, Linnaeus 1758), with its peculiar life cycle, represents a fish species of great interest. From a naturalistic perspective, through restocking campaigns to encourage migration of eels, financed projects within the EU LIFE program have supported the purpose of species conservation. Nevertheless, many factors continue contributing to the decline of eel populations, and among them, environmental conditions and health issues play an important role. The skin and the cutaneous mucus represent the first line of defense of the host against infections. The development and application of non-invasive methods to study fish secretions, including the cutaneous mucus and admixed exfoliated cells, can significantly contribute to obtain an overall picture of the fish’s health status, thus contributing to safeguarding this endangered fish species. With this preliminary investigation, two non-invasive methods were applied for the first time to a free-living European eel population. The antibacterial capacity of the mucus and the cellular components obtained through skin scrapings were investigated. Both methods were revealed to be effective, providing additional contributions to the eels’ health status.

## 1. Introduction

Mucus constitutes a natural, physical, biochemical, and semipermeable barrier that allows the exchange of nutrients, water, gases, odors, hormones, and gametes [1], and is the result of a dynamic secretion that is continuously renewed, preventing the adhesion and stable colonization of potentially infectious microorganisms. The composition of mucus largely depends on the physiological and health condition of the organism [2].

The antimicrobial capacity of mucus seems to derive from its mechanical and biochemical properties; currently, the available information is scarce and limited to a few fish species, but it appears that this activity is directly influenced by the fish species and the pathogen species that infects it [3]. The adhesion of bacteria to mucosal surfaces depends on the state of the mucus itself and the situation of the surrounding environment [4]. Mucus has the ability to combat the colonization of pathogenic agents, but also to tolerate the colonization of commensal or saprophytic microbial agents. The analysis of skin mucus is considered a method to evaluate the physiological response of fish facing stress and environmental challenges and is considered a less invasive method compared to blood analysis [4]. Concerning bacteria, there are species which can be considered opportunistic or primary pathogens. The eel is highly sensitive to *Aeromonas hydrophila*, an opportunistic bacterium which could become pathogenic when there is a decrease in the host’s immune defenses, in association with a suboptimal environment for the host’s life and the intrinsic virulence of the pathogen itself. When these situations do not occur, these bacteria remain in symbiosis with the fish without causing any harm [5]. *Aeromonas salmonicida* is the oldest known primary pathogen of fish distributed worldwide, both in freshwater and marine waters. The *A. salmonicida* subspecies *salmonicida* is known as a pathogen of salmonid fish, in which it causes furunculosis. The development of aquaculture and intensive rearing has led to the isolation of new strains of the bacterium belonging to “atypical” subspecies [6], which have been recognized as pathogenic bacteria also for eels [5].

The development of aquaculture has led to the isolation of new pathogenic bacterial strains, but one of the first barriers against exogenous hazards in fish is the mucus, secreted by goblet cells in the skin, that serves as an active key defense mechanism, and is involved in the continuous exchange of ions, hormones, waste compounds, and protection against potential pathogenic microorganisms. Cutaneous mucus is secreted by goblet cells present in the epidermal layer of the fish skin. These cells in fish constitute a living cellular layer that is capable of continuously secreting and absorbing products such as ions, hormones, and waste compounds, as well as pathogens and chemical substances [3]. There are several cytological sampling techniques applicable in fish tissues, which are borrowed from their use on other animals. Among them, smears, swabs, and scrapings are often used, in conjunction with classic wet-mount preparations, on erosive and ulcerative lesions and on tissues such as epithelia that give off cells, while fine needle aspiration biopsies are used to guide diagnosis in the presence of nodules or masses [7].

With this preliminary work, we aimed to apply to a free-living European eel (*Anguilla anguilla*, Linnaeus 1758) population sample two non-invasive methods, to study the antibacterial capacity of the cutaneous mucus and the cellular components embedded within this biological matrix.

## 2. Materials and Methods

### 2.1. Sample Collection

The sampling was carried out during field activities within the EU Life Biodiversity Project (LIFE19 NAT/IT/000851) “Urgent measures in the Eastern Mediterranean for the long-term conservation of endangered European eel (*Anguilla anguilla*).” At the end of the non-invasive procedures’ application, the eels were released in their natural environment. Animals were collected from the Comacchio lagoon (Emilia-Romagna region, Italy).

The sampling of the eel population was carried out in November 2024 on 24 females with an average weight of 1.438 g and an average length of 87 cm. For each individual, a mucus sample was taken for the antibacterial capacity test and after it, a skin smear on a slide was taken for cytological examination. The mucus was collected directly after the animals were extracted from the water. With the aid of a slide, the mucus was removed by gently rolling the slide over the skin two–three times following the lateral line of the fish in the cranio–caudal direction. The procedure of sampling was carried out on both sides of the fish. The mucus was then collected in numbered sterile 1.5 mL tubes. During all sampling, the procedure was performed avoiding any contact with unwanted areas such as the operculum, ventral-anal, and caudal zones, thus avoiding urinogenital contamination and intestinal secretion contact. It was also crucial not to rub with too much force on the body surface of the animal, as this could lead to the appearance of epidermal lesions, resulting in contamination of the samples with blood and epithelial cells [4]. Once the samples arrived at the laboratory on the same day, the mucus was centrifuged at 14,000× *g* for 15 min at 4 °C. The supernatant was then collected, avoiding the cellular pellet on the bottom, aliquoted, and stored at −80 °C according to the following reference [2]. At the time of testing, the mucus was then thawed and processed as a pool of mucus, merging the samples collected from the eel population.

### 2.2. Bacterial Growth

Twenty-four hours before each test, solutions with the bacterial strains *A. hydrophila* and *A. salmonicida*, originally isolated at 25 °C from two koi carps (*Cyprinus carpio*) showing health problems during the summer season, were cultured in 3 mL of Tryptic Soy Broth (TSB, Oxoid, UK); the broths were then incubated at 25 °C under agitation overnight, and before starting the test, the bacterial testing solutions were standardized at an optical density (OD) value of 0.2 according to the method previously described [2].

### 2.3. Antibacterial Plate Assay

The effect of skin mucus on bacterial growth was determined by co-culture challenges in flat-bottomed 96-well plates according to a method previously described with minimal changes [2]. Briefly, each test well (T) was loaded with 50 μL of bacterial suspension plus 100 μL of skin mucus and 50 μL of culture media to obtain a 200 μL final volume. Mucus controls (M) without bacterial suspension were prepared by adding 100 μL of culture media and 100 μL of skin mucus. Bacterial growth without mucus (B) was prepared by adding 50 μL of bacterial suspension and 150 μL of culture media. Blanks (control bacterial growth without bacteria and mucus—TSB) were prepared by adding 200 μL culture media. Plates were incubated at 25 °C in agitation overnight. The absorbance of the bacteria was measured at λ = 630 nm every 60 min for 8 h in flat-bottomed 96-well plates. Samples were done in triplicate (methodological replicates) and the antibacterial capacity assay was repeated twice (biological repeats).

The average absorbance of mucus controls (M) without bacteria was subtracted from the absorbance from co-culture (bacteria plus skin mucus) samples (T).

### 2.4. Cytological Analysis

Concerning cytology, a skin smear by scraping was carried out from each individual at the end of the mucus sampling. In this case, it consisted of rubbing the skin of the fish gently with a beveled glass slide in the lateral and cranial areas, in order to stimulate the secretion of additional mucus; then, with the help of another slide, the mucus was spread on it, and the slides were then carefully placed in a slide holder. The cytological slides, once dried, were subjected to May–Grünwald Giemsa (MGG) (Histoline, Milan, Italy) staining, covered with cover slips, and examined under an optical microscope (Nikon, Eclipse E600, Tokyo, Japan).

## 3. Results

The results of tests on the antibacterial activity of eel skin mucus were shown as bacterial growth curves (increased absorbance per unit of time) and as percentage of inhibition with respect to bacterial growth, when cultured in the presence of the cutaneous mucus.

**Figure 1:**
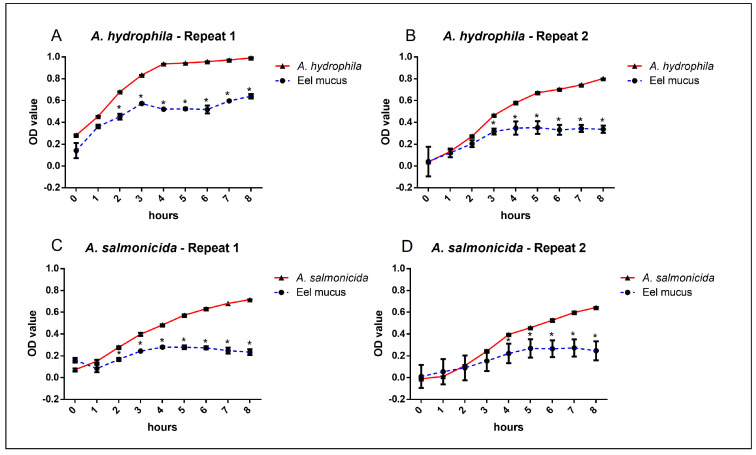
Skin mucus bacterial growth. The bacterial growths are shown for *A. hydrophila* (**A**,**B**) and *A. salmonicida* (**C**,**D**) co-cultured for 8 h with European eel (*Anguilla anguilla*) skin mucus (blue lines) in comparison to bacterial growths without mucus (red lines). Data are shown as mean ± standard deviation of the mean (*n* = 3). An asterisk (*) indicates statistically significant differences between bacterial growth with and without mucus (*p* < 0.05).

The bacterial growth curves showed that eel mucus was able to significantly reduce the growth of both *A. hydrophila* and *A. salmonicida* (Figure 1).

**Figure 2:**
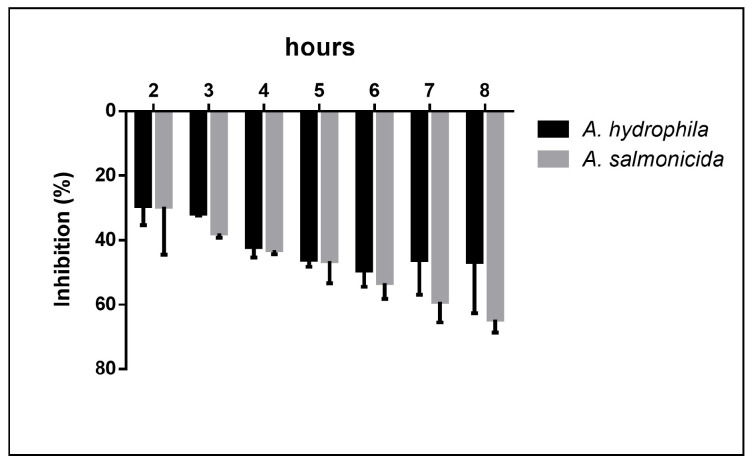
Inhibition rate. Bacterial inhibition rates are shown for *A. hydrophila* (black) and *A. salmonicida* (grey) co-cultured for 8 h with European eel (*Anguilla anguilla*) skin mucus. Data are shown as the mean ± standard deviation of the mean of two biological repeats. No statistically significant differences were observed between the inhibition of the two tested bacteria (*p* > 0.05).

Both biological repeats of each bacterial strain showed the same reduction trend, with significant bacterial reduction growth value between 2 and 8 h when cultured with eel mucus. The highest growth reduction was reached at the 8th hour for *A. hydrophila* with a 64.6% reduction, while the reduction of *A. salmonicida* growth peaked at the 6th hour (49.4%), maintaining a high reduction value for the following 2 h (46.7%). No significant differences were observed between *A. hydrophila* and *A. salmonicida* growth reduction during the 8 h tests (Figure 2).

**Figure 3:**
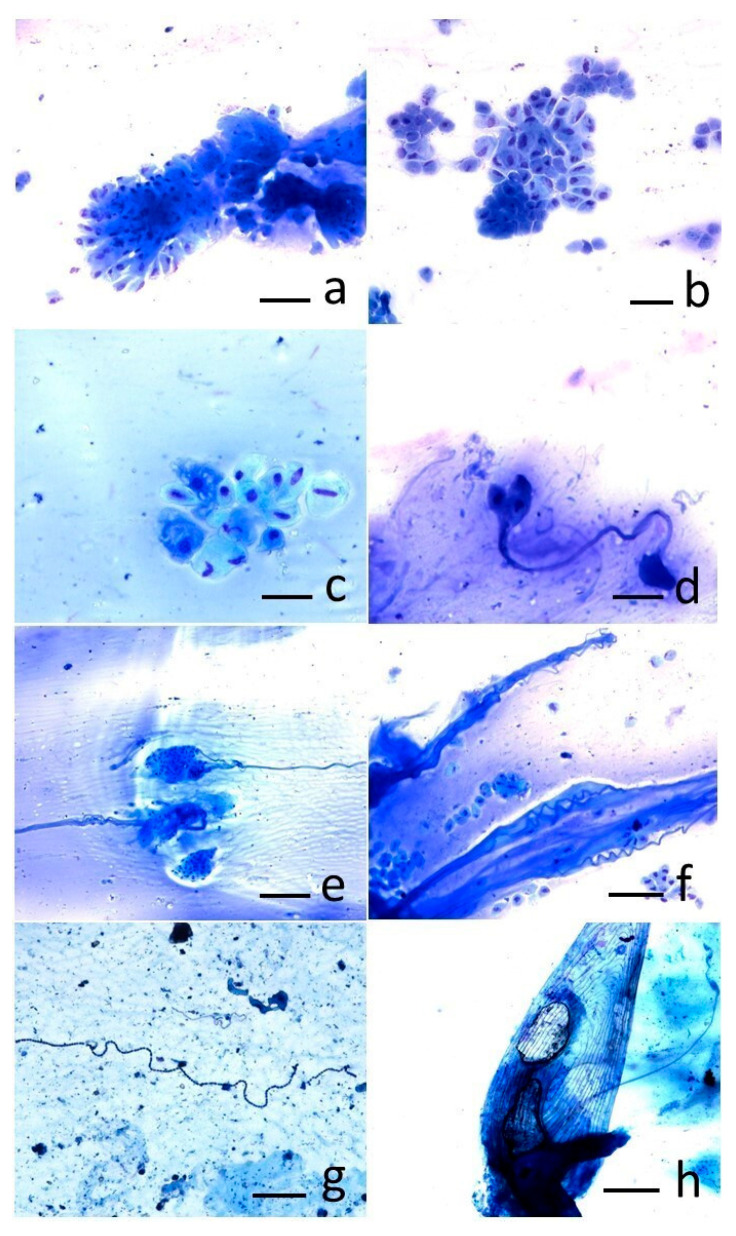
European eel. Skin scraping cytology from cutaneous mucus. MGG stain. (**a**) Aggregates of epidermal cells, scale bar: 300 µ; (**b**) clusters of goblet cells, scale bar: 100 µ; and (**c**) at higher magnifications, intracytoplasmic vacuoles storing mucus are visible, scale bar: 50 µ. (**d**) Strands of mucus secreted by a small group of goblet cells, scale bar: 300 µ; (**e**,**f**) Curshmann’s spiral-like mucus produced by goblet cells, scale bar: 300 µ; (**g**) bacterial polymicrobism, scale bar: 500 µ; (**h**) an intact scale, scale bar: 500 µ.

Concerning cytological samples, two cellular populations represented the main results of sampling, the epidermal and goblet cells. The epidermal cells, often arranged in clusters, showed polygonal morphology and well-defined cell boundaries (Figure 3a). Goblet cells, consisting of smaller secretory cell clusters, were also detected (Figure 3b). Within the goblet cells, cytoplasmic vacuolations, indicative of production of mucous material stored and not yet secreted, were often detected (Figure 3c). As result of goblet cell secretion, some mucus strands were also visible (Figure 3d); sometimes the abundant amount of mucus was present as spirals (Figure 3e,f). Some smears were characterized by the presence of numerous bacteria of polymorphic appearance (cocci and bacilli), in absence of inflammatory cells (Figure 3g). Occasionally, single and intact scales, characterized by an elliptical shape and consisting of concentric circular lines typical of eels, were present as well (Figure 3h). Overall, these findings photographed the physiological status of the skin of sampled eels.

## 4. Discussion

Mucus is primarily composed of water and jellifying macromolecules, including mucins and other glycoproteins [8]. The carbohydrates in mucins can act as receptors for microorganisms, playing a crucial role in both the expulsion of the pathogen and its settlement and invasion [1]. Moreover, a wide variety of structural molecules are present in mucus, including lectins or actin, enzymes such as lysozyme, phosphatases, esterases, or proteases, and other stress-related proteins such as heat shock proteins, transferrin, and histones; antimicrobial peptides constitute an innate defense mechanism against pathogens [3]. Lysozyme is probably the most potent bacteriolytic enzyme as it acts directly on Gram-positive bacteria and the inner layer of peptidoglycans of Gram-negative bacteria following the breakdown of the outer wall by complement and other enzymes [9]. The bacteriolytic activity of this enzyme varies greatly among different fish species, and this could influence the varying resistance of fish to bacterial pathogens or depend on the abundance/diversity of bacteria in aquatic environments [1].

The analysis of skin mucus is considered a method to evaluate the physiological response of fish in facing stress and environmental challenges, and is a less invasive method compared to blood analysis, which could cause skin lesions that increase the likelihood of increase in stress itself and bacterial and fungal infections [4]. The antimicrobial activity of mucus seems to derive from its mechanical and biochemical properties; currently, the available information is scarce and limited to a few fish species, but it appears that this activity is directly influenced by the fish species and the pathogenic species that infects it [3]. In European sea bass (*Dicentrarchus labrax*), in which an infection by *Vibrio anguillarum* was simulated, increase of mucus secretion with a loss of soluble proteins was observed, indicating changes in protein turnover to combat the infection [2]. The antimicrobial and antiproliferative activity of skin mucus has also been studied in *Dasyatis pastinaca* (Linnaeus, 1758), providing information on the development and identification of chemotherapeutic agents to overcome microbial and tumor chemoresistance [10]. A comparative analysis was conducted on the humoral immunity of the mucus of five different species of commercially important marine teleosts: gilthead sea bream (*Sparus aurata*), European sea bass (*Dicentrarchus labrax*), umbrine (*Umbrina cirrosa*), common dentex (*Dentex dentex*), and dusky grouper (*Epinephelus marginatus*) [1]. Furthermore, the antimicrobial activity of mucus had also been analyzed in the epidermal mucus of various fish species in the North Atlantic, including the Arctic char (*Salvelinus alpinus*), the brook trout (*S. fontinalis*), the koi carp (*C. carpio subsp. koi*), the striped bass (*Morone saxatilis*), the haddock (*Melanogrammus aeglefinus*), and the hagfish (*Myxine glutinosa*), with the aim of better understanding their functions and identifying potential fish species which produce antimicrobial compounds for applications in animal and human health [9].

Hitherto, European eel mucus has only poorly been investigated; however, molecules isolated from eel mucus showed potential antibacterial activity [11,12]. Furthermore, some studies showed an increased resistance to bacteria, including *A. hydrophila*, in association with the boosting of several innate- and adaptive-immune parameters including specific activities and genes expressed in mucus [13,14,15]. However, to the best of our knowledge, the direct antibacterial capacity of free-living European eel skin mucus has not yet been investigated.

In this study, a non-invasive approach was used to test the antibacterial capacity of European eel skin mucus, showing its ability to significantly counteract the proliferation of two bacteria, the opportunistic pathogen *A. hydrophila* and the primary pathogen *A. salmonicida*. Mucus’s mechanical and biochemical characteristics appear to be the source of its antimicrobial activity. The condition of the mucus itself and the surrounding environment affect how well bacteria adhere to mucosal surfaces [4]. Mucus can tolerate the colonization of commensal or saprophytic microbial organisms, in addition to preventing the colonization of harmful agents. Among the deadliest bacteria, those of the genus *Aeromonas* play a crucial role. Motile *Aeromonas* spp. can act as saprophytes, living in the environment and multiplying on decomposing fish or other organic material present in the water. Furthermore, these bacteria can be associated with algae, biofilms, and zooplankton and are natural components of the intestinal flora of many vertebrates and invertebrates. However, in appropriate conditions, such as high temperature and organic load, their number can increase and their opportunistic nature can prevail, showing their pathogen potential. Three species of motile *Aeromonas*, namely *A. hydrophila*, *A. caviae*, and *A. veronii* biovar sobria, can cause a disease called Motile Aeromonas Septicemia (MAS) [16]. Motile *Aeromonas* appear to be primarily transmitted horizontally by direct contact with infected fish or contaminated water and material. Asymptomatic carriers likely spread the bacterium in their feces, while sick individuals may release the bacterium from open lesions and are often preyed upon by other fish. The absorption of the bacterium can occur orally or dermally, resulting in the alteration of mucosal/skin defenses [16]. In particular, the ability to adhere has been studied for pathogenic *A. hydrophila* in eel mucus and associated to specific genes [17,18,19]. As a matter of fact, *A. hydrophila* is a main pathogenic bacterium for several eel species, including the European eel, and its infection has been reported in both farmed and wild populations, whereas this bacterium has been isolated from healthy eels more rarely [20,21,22]. The atypical subspecies of *Aeromonas salmonicida* are recognized as primary pathogenic bacteria for eels, as well as agents of carp erythrodermatitis and pathogens of other non-salmonid species [5]. Among the virulence factors of *A. salmonicida* that cause clinical signs, such as skin ulcerations and hemorrhages, there is the type 3 secretion system, which functions to interfere with the immune response within the host and suppress the inflammatory response. In the American eel (*Anguilla rostrata*) and the Japanese eel (*A. japonica*), the disease is characterized by small, discolored spots on the skin that can turn into deep ulcers ranging from 5 to 30 mm in diameter, often involving the underlying muscle tissue. Furthermore, branchial lesions were associated with the presence of edema, necrosis, inflammatory cells, and isolated bacteria or bacterial microcolonies [6].

However, experimental challenge in the European eel showed a higher resistance of this species to *A. salmonicida* infection. In fact, few clinical signs and mortality were induced by *A. salmonicida* intraperitoneal and intramuscular injection; on the other hand, the European eel was shown to be resistant to *A. salmonicida* when challenged by immersion, suggesting the ability to block the bacterium through physical barriers [23]. Accordingly, our study showed a high antibacterial capacity associated with the mucus against *A. salmonicida*. Although our results confirm the antibacterial effectiveness of *A. anguilla* skin mucus, the present study did not include a chemical characterization of its components. Consequently, the specific molecules responsible for the observed antimicrobial activity remain unidentified. Further studies employing proteomic and biochemical analyses will be essential to isolate and characterize the active compounds that may contribute to the inhibition of bacterial growth. Understanding the chemical profile of eel mucus would enhance our interpretation of its role in innate immunity and protection against pathogens.

Fish mucus plays a critical role in the innate immune defense of teleosts, providing both a physical barrier and a chemically active interface against a wide range of aquatic pathogens [24,25]. It is composed of a complex array of antimicrobial molecules, including antimicrobial peptides (AMPs), lysozymes, proteases, lectins, complement proteins, and immunoglobulins, all contributing to its potent antibacterial properties [8,26]. Mucus from Atlantic salmon (*Salmo salar*) has shown antimicrobial effects against *Vibrio anguillarum*, *Aeromonas salmonicida*, and *Moritella viscosa* [27]. Similarly, mucus from Nile tilapia (*Oreochromis niloticus*) has demonstrated inhibitory activity against *Aeromonas hydrophila* and *Pseudomonas fluorescens* [28]. Likewise, mucus from common carp (*Cyprinus carpio*) has been reported to inhibit *Flavobacterium columnare*, *Aeromonas hydrophila*, and *Edwardsiella tarda* [29]. Regarding catadromous species, European eel mucus antifungal activity has been assessed against *Aspergillus awamori*, *Colletotrichum falcatum*, and *Fusarium oxysporum*, with effective results [30]. Recently, the bacterial inhibitory capacity of fish mucus has raised significant interest in relation to clinically significant pathogens; in fact, the acidic extracts of epidermal mucus from *Clarias batrachus* exhibited antimicrobial activity against a range of human pathogens, including *Bacillus licheniformis*, *Vibrio cholerae*, *Escherichia coli*, *Klebsiella pneumoniae*, *Staphylococcus aureus*, *Salmonella typhi*, and *Pseudomonas aeruginosa* [31]. In a study involving an intraperitoneal challenge on European eels, researchers evaluated the bactericidal properties of skin mucus against three opportunistic marine pathogens: *Tenacibaculum soleae*, *Vibrio anguillarum*, and *Photobacterium damselae* subsp. piscicida. The findings indicated that eels exposed to *V. anguillarum* showed enhanced bactericidal activity in their mucus 48 h post-challenge when compared to unexposed controls. Notably, mucus from fish challenged with *P. damselae* displayed a marked increase in antimicrobial activity at all examined time points relative to other treatment groups. Based on these results, the authors suggested that the mucus’s bactericidal effect likely results from a combination of multiple antimicrobial molecules working synergistically against pathogens [32]. Accordingly, the present study aimed to broaden the spectrum of pathogens targeted by mucus antimicrobial capacity by evaluating the antibacterial activity of European eel mucus against *Aeromonas hydrophila* and *Aeromonas salmonicida*. To the best of our knowledge, this is the first report investigating the effect of eel mucus on these two bacterial species. The choice of *A. hydrophila*, an opportunistic pathogen, and *A. salmonicida*, a primary pathogen, was intended to assess the mucus response against bacteria with differing pathogenic profiles.

The antibacterial action of fish mucus involves multiple mechanisms, including membrane disruption, oxidative damage, inhibition of bacterial growth and adhesion, and modulation of quorum sensing signals [33,34]. The efficacy of mucus against different bacterial species varies according to factors such as fish species, health status, environmental stressors, and pathogen load [35,36]. In the present study, fish mucus exhibited inhibitory activity against *A. hydrophila* and *A. salmonicida* isolates, confirming its protective role. These observations align with previous research indicating that mucus defenses are highly adaptable and form a key part of the fish’s response to environmental challenges and infection pressure [37,38]. Given the effectiveness of the method in evaluating the antibacterial activity of skin mucus against *A. hydrophila* and *A. salmonicida*, this approach could be successfully extended to other bacterial species. Such applications could help assess the broad-spectrum antimicrobial potential of eel mucus and support comparative studies among different fish species or environmental conditions. Additionally, testing the response against emerging or multidrug-resistant strains could provide valuable insights for developing alternative antimicrobial strategies in aquaculture and conservation programs.

Cytology is a safe, inexpensive, rapid tool which can be effectively employed in superficial tissues as skin [7]. Cytological specimens should be collected using both brush and touch techniques. From the skin of healthy fish, mucus, squamous epithelial cells, and occasional scales with or without pigment can be sampled. Some parasites in low numbers can occasionally be found as commensal elements. Moreover, bacterial aggregates can be present: they may reflect post-mortem overgrowth or, in the presence of a monomorphic bacterial population, associated with the presence of inflammatory cells and damaged scales, may suggest an infection [7]. Lesions on the fish’s skin are typically the result of a harmful stimulus that can be responsible for cellular degeneration, erosion, and ulceration, or of proliferative stimuli that are responsible for a hyperplastic condition. The initial factors can be masked by secondary opportunistic infections; these opportunistic agents can still be subject to cytological sampling and include hyphae of oomycetes. The fish epidermis is composed of a stratified epithelial layer of non-keratinized elements of varying thickness that changes based on species, age, site, and reproductive status [7]. Goblet cells differentiate from epithelial cells in the lower layers of the epidermis; immature ones have a rounded shape, but as they become mature, they tend to flatten laterally and increase in size as they move towards the surface of the epidermis. The nucleus and organelles shift to a basal position, and the cell membrane breaks at the apical site, allowing secretions to be released. The abundance and size of goblet cells can vary in different regions of the body [39]. In the current study, the two most represented cellular populations of the cutaneous layer, the epidermal cells and the goblet cells, represented the main fruit of sampling. The morphology of the cellular aggregates and the cells themselves were interpreted as expression of a healthy skin status. In addition to these findings, the cutaneous mucus arranged in strands recalled the findings comparing two cytological methods applied to bronchoalveolar lavage in horses affected by severe asthma [40]. In our study, the mucus strands were arranged to form spirals, which resembled Curshmann’s spirals resulting from excessive mucus production in human patients suffering from asthma or chronic bronchitis [41]; however, in our study, these findings were considered a normal expression of the abundant amount of mucus physiologically produced by the eel skin. Moreover, the polymorphic bacteria, morphologically consistent with bacilli and cocci, in the absence of an inflammatory reaction, have been considered the expression of environmental polymicrobism.

## 5. Conclusions

The skin and its secretions, such as mucus, represent one of the most important mechanisms of defense against infections by pathogens. The cutaneous mucus and the cells entrapped within this matrix can be suitable non-invasive biomarkers to monitor the health status of farmed and free-living fish populations, assessing the efficacy of their defense barrier biomarkers [3]. However, the antimicrobial activity of mucus seems to be directly influenced by the fish species and the pathogenic species that infects it, so specific studies need to be conducted to assess the antimicrobial capacity of a specific species against certain pathogens [3].

With this preliminary study, a non-invasive method to assess the antibacterial mucus capacity of an eel population for the first time was successfully applied. The tested eel mucus was able to significantly reduce the bacterial growth of both the tested bacteria, suggesting that under ideal conditions healthy European eels can, to some extent, counteract *A. hydrophila* and *A. salmonicida* infections.

In parallel to the antimicrobial capacity evaluation method employed, cytological sampling by skin smear has proved to be a useful non-invasive tool as well, having allowed the identification of the two most represented cell populations of eel skin, the epidermal lining cells and the goblet cells. Additionally, mucus secretion was recognized as forming characteristic extracellular strands, often arranged in spirals, as an expression of the abundant amount of this matrix, typical of the eel skin.

As several environmental conditions, in addition to infections, can also affect the antibacterial capacity of mucus, the successfully applied methods can find a wide application to understand if different environmental circumstances, including feeding and breeding situations as well as different geographical realities, may affect the natural ability of the European eel to counteract bacterial infections.

Although this study is only a preliminary approach, we recommend a wider use of both these complementary non-invasive microbiological and cytological methods, which can provide relevant information on the health and welfare status of the fish populations analyzed, indirectly contributing to the conservation of the species.

## Data Availability

Data are available upon request.
